# Depletion of primary cilia from mature dentate granule cells impairs hippocampus-dependent contextual memory

**DOI:** 10.1038/srep34370

**Published:** 2016-09-28

**Authors:** Soyoung Rhee, Gregory W. Kirschen, Yan Gu, Shaoyu Ge

**Affiliations:** 1 Program in Molecular and Cellular Pharmacology, State University of New York at Stony Brook, Stony Brook, New York, USA; 2 Medical Scientist Training Program, State University of New York at Stony Brook, Stony Brook, New York, USA; 3 Center of Stem Cell and Regenerative Medicine, Zhejiang University School of Medicine, Hangzhou, China; 4 Department of Neurobiology and Behavior, State University of New York at Stony Brook, Stony Brook, New York, USA

## Abstract

The primary cilium, a sensory organelle, regulates cell proliferation and neuronal development of dentate granule cells in the hippocampus. However, its role in the function of mature dentate granule cells remains unknown. Here we specifically depleted and disrupted ciliary proteins IFT20 and Kif3A (respectively) in mature dentate granule cells and investigated hippocampus-dependent contextual memory and long-term plasticity at mossy fiber synapses. We found that depletion of IFT20 in these cells significantly impaired context-dependent fear-related memory. Furthermore, we tested synaptic plasticity of mossy fiber synapses in area CA3 and found increased long-term potentiation upon depletion of IFT20 or disruption of Kif3A. Our findings suggest a role of primary cilia in the memory function of mature dentate granule cells, which may result from abnormal mossy fiber synaptic plasticity. A direct link between the primary cilia of mature dentate granule cells and behavior will require further investigation using independent approaches to manipulate primary cilia.

Cilia are hair-like structures composed of microtubules, which can be classified as either motile or primary based on their movement and microtubule structure^1^. In the mammalian brain, each of various cell types, such as stem/progenitor cells, neurons and glial cells, expresses a single primary cilium, which is generally involved in cell signaling^2^,^3^. Several studies of neuronal primary cilia have focused on cell proliferation, and a few recent ones on neuronal migration during embryonic brain development^4^,^5^,^6^. We recently found that almost all developing adult-born dentate granule cells (DGCs) form primary cilia immediately preceding the period of glutamatergic synapse formation^7^, corresponding to their electrophysiological and functional maturation^8^. Moreover, these primary cilia are essential for proper integration of DGCs into the existing circuit^9^. That most adult and embryonically born DGCs retain primary cilia led us to speculate that primary cilia may be important in hippocampus-dependent behavior.

Moreover, mutations in ciliary proteins that cause structural or functional abnormalities are implicated in a wide variety of diseases known as ciliopathies, and defects in primary cilia can lead to neurological impairments such as intellectual developmental disorders and cognitive deficits^10^,^11^,^12^. Previous research has suggested that the pathological phenotypes of ciliopathies might be due to dysfunction in primary cilia-associated signaling pathways during development^5^,^13^,^14^. However, whether neuronal primary cilia have any direct physiological role in regulating neuronal activity or behavior is largely unknown.

Here, we examined the physiological and behavioral function of primary cilia in mature DGCs. We conditionally ablated intraflagellar transport (IFT) genes, IFT20 or expressed a dominant negative form of Kinesin family member 3A (Kif3A) under the control of the CaMKII promoter using adeno-associated viruses (AAV). We found that ablation of IFT20 in mature DGCs led to impaired hippocampus-dependent contextual memory. We further found that IFT20 ablation or Kif3A disruption in mature DGCs enhanced long-term potentiation (LTP) induced at mossy fiber (MF) synapses of these cells.

## Materials and Methods

### Mice

Conditional IFT20 mutant mice were kindly provided by Dr. Gregory J Pazour at the University of Massachusetts Medical School^15^. All mice used in experiments were housed under a 12-hour light/dark cycle. All animal procedures were conducted in accordance with the institutional animal guidelines and were approved by the ethical committee of the State University of New York at Stony Brook.

### Manipulation of mature dentate granule cells

High titer engineered AAV9 under the CaMKII promoter was purchased from the inventory of vector core facility from University of Pennsylvania. AAV9-DIO-dnKif3A-dTomato was produced as described previously^9^. High titer AAVs were stereotaxically injected into the dentate gyrus of ~5–6 week-old adult mice. To allow for efficient viral transduction and maturation of potentially labeled newborn DGCs^16^, mice were used for experiments 14 days after AAV injection.

### Immunohistochemistry and quantification

Mice were anesthetized and transcardially perfused with PBS and 4% PFA. Fixed brains were sliced by a sliding microtome, creating 40 μm thick coronal brain sections.

Prior to primary antibody incubation, brain sections were blocked with 10% donkey serum in 0.25% Triton-PBS for 1 hour. Next, sections were incubated overnight at 4 °C with diluted primary antibodies (rabbit anti-adenylyl cyclase III 1:300, Santa Cruz Biotechnology, goat anti-GFP 1:500, Rockland, goat anti-DCX 1:500, Santa Cruz Biotechnology, mouse anti-parvalbumin, 1:1000, Sigma). The next day, brain sections were incubated in diluted secondary antibodies solution (mouse anti-goat AlexaFluoro 488 1:1000, Jackson Labs, Donkey anti-rabbit CY-3 1:1000, Jackson Labs, donkey anti-rabbit AlexaFluoro 647 1:1000, Jackson Labs, donkey anti-mouse Cy-3, 1:1000, Jackson Labs) for 2 hours at room temperature. Sections were mounted on slides using DAPI-containing mounting media to stain nuclei. All images were acquired on an Olympus FV1000 confocal system. For DCX + cell quantification, randomly selected, 40 μm-thick, z-stacked images were collected across the entire anterior/posterior axis of the dentate gyrus (field side 320 μm × 320 μm; 60 fields from 3 control mice, 72 fields from 4 IFT20 (−/−) mice), and 2 μm guard zones were used to avoid edge artifacts. Dendrite morphology was analyzed using the Sholl analysis function of the NeuronJ plug-in for ImageJ.

Cilia were identified and related to their cell of origin for quantification using an anatomical- and landmark-based approach^4^,^9^. Briefly, each mature DGC lines up perpendicular to the GCL and extends a single primary cilium radially in the same direction and in the immediate vicinity of its primary apical dendrite. Moreover, the primary cilium, which is thickest at its base, arises from the mother centriole, lying immediately superior to the nucleus in the DGC. Based on these features, 3-dimensionally reconstructed z-stacked images were used to identify all cells expressing primary cilia (DAPI + /ACIII+), as well as virus-labeled cells expressing primary cilia (by overlaying GFP).

Analysis of parvalbumin and GFP co-localization was conducted by taking 40 μm-thick, z-stacked images across the entire anterior/posterior axis of the dentate gyrus (field side 320 μm × 320 μm). Co-localization was confirmed by overlaying GFP in 3-D reconstructed images of parvalbumin + cells in subgranular zone and hilus of the dentate gyrus.

### Contextual fear conditioning (CFC)

We conducted the procedure for CFC as we have previously described^16^. Mice were placed in a fear-conditioning chamber consisting of transparent front and back walls, stainless steel sidewalls and a stainless steel shock grid floor (18 × 18 × 30 cm, Coulbourn). In fear context A, 70% ethanol was used to remove any odor for the context before each experiment. During training in context A, mice were allowed to explore the context for 2 minutes before a 30 second tone (2800 Hz, 85 dB) followed by a 2 second foot shock (0.5 mA). Mice were removed from the conditioning chamber 30 seconds after the foot shock and transferred back to their home cages. A probe test for contextual fear memory was conducted 24 hours after training. Mice were re-exposed to context A without tone or foot shock, and freezing levels during a 5-minute period were measured. A probe test for toned fear memory was conducted 4 hours after the previous probe test. Mice were placed into a novel context B, which consisted of white plastic surrounding walls and a covered floor without an odor cue. A tone was presented for the last 3 minutes of the 5-minute probe test to evaluate tone-conditioned fear memory, and freezing level during this probe test was measured. Mice movements were recorded using Freeze Frame software, and freezing levels were analyzed by Freeze View software with 1-second minimum bout duration.

### Pre-exposure contextual fear conditioning (PECFC)

PECFC was conducted to probe pattern memory encoding on the basis of partial cue information from a fear context to which an animal was pre-exposed. We used an experimental paradigm based on previous work^17^. Briefly, on day 1, mice were allowed to freely explore in a conditioning chamber, same as described in CFC test, for 10 minutes. Twenty-four hours after the pre-exposure, mice were re-exposed to the context and received a foot shock 10 seconds after entering the context. A probe test was conducted on day 3 by analyzing the freezing level of mice during a 5-minute exposure period to examine the expression of a previously formed fear memory based on partial cues from the pre-exposed context.

### Contextual Fear Discrimination Test

We used a contextual fear discrimination test adapted from previous work^18^. On day 0, mice received a foot shock at context A to generate a contextual fear association. This context contains a stainless steel grid floor, transparent front and back walls with a mild alcohol scent for the olfactory cue. This context was cleaned with 70% ethanol before each mouse was placed in the context. For fear memory learning, mice received a 2 second single foot shock (0.5 mA) at 185 seconds after placement into the chamber. Mice were removed from the chamber and returned to home cages 15 seconds after the foot shock termination.

For the next 8 consecutive days, mice were exposed to context A (fear context) and a similar context, context B (safe context in which no shock was delivered). Context B consisted of a stainless steel grid floor as in context A, but contained black and white striped front and back walls and lacked an olfactory cue. This context was cleaned with odorless non-alcoholic antiseptic solution before each mouse was placed in the context. Mice were allowed to explore freely for 180 seconds without any shock. The order of exposure on each day was AB/AB/BA/BA/AB/BA/AB and there was a 4-hour gap between exposures to each context.

All mice movements were recorded through FreezeFrame software and percentage of freezing was analyzed by FreezeView software with 1 second of minimum bout duration. Freezing levels during the first 180 seconds of each session were averaged for each group and used to calculate discrimination ratios;

Discrimination Ratio = (Freezing Context A – Freezing Context B)/(Freezing Context A + Freezing Context B).

### Spatial novelty recognition test

We performed the spatial novelty recognition test similar to what has been previously described^19^. On days 1–3, mice were habituated to an open arena (50 cm^3^ opaque box) lacking objects. On day 4, mice were exposed to two identical objects arranged in one positioning pattern for 10 minutes. On day 5, one of the two objects was moved to a novel location. Object location zones were defined and animal paths were recorded using EthoVision XT video tracking software.

### Elevated Plus Maze (EPM)

The EPM is a paradigm used for testing anxiety-like behavior in rodents^20^. The apparatus used for the EPM has two open arms and two closed arms surrounded by transparent plastic walls, and stands 50 cm above the floor. To test anxiety-like behavior, mice were placed at the center of the apparatus and allowed to explore freely for 5 minutes. The time spent in each arm and the center was measured and analyzed, with longer time spent in the open arms interpreted as corresponding to lower anxiety-like states.

### Light/Dark transition test

The light/dark transition test measures anxiety-like behavior associated with rodents’ innate light avoidance. The apparatus has two connecting compartments- a light compartment that has transparent plastic walls and was illuminated during experiment, and a dark compartment that is covered with black and opaque plastic walls. Mice were placed in the dark compartment and allowed to freely explore both compartments for 10 minutes. Latency to enter the light compartment and time spent in each compartment were measured.

### Electrophysiology

Preparation of brain slices and electrophysiological recordings was performed as previously described^16^,^21^. To examine synaptic plasticity of the MF tract, we placed a stimulating electrode at the MF tract near the dentate gyrus and a recording electrode in area CA3. Field excitatory postsynaptic potentials (fEPSPs) were recorded with an intensity that produced a half maximal response. High frequency stimulation (HFS) (100 stimuli at 100 Hz) was delivered to induce synaptic plasticity. The average of fEPSPs slope in every 4 sweeps was analyzed. LTP was determined by comparing the averaged fEPSPs slope of the baseline (-10-0 minutes) and the last 10-minute (50–60 minutes).

### Statistical analysis

Data were analyzed using two-tailed unpaired Student t-tests, repeated measures ANOVAs, and two-way ANOVAs. The cut-off for statistical significance was set at a p value of less than 0.05. All data are presented as mean ± SEM.

## Results

### Depletion of primary cilia from mature dentate granule cells

**Figure 1:**
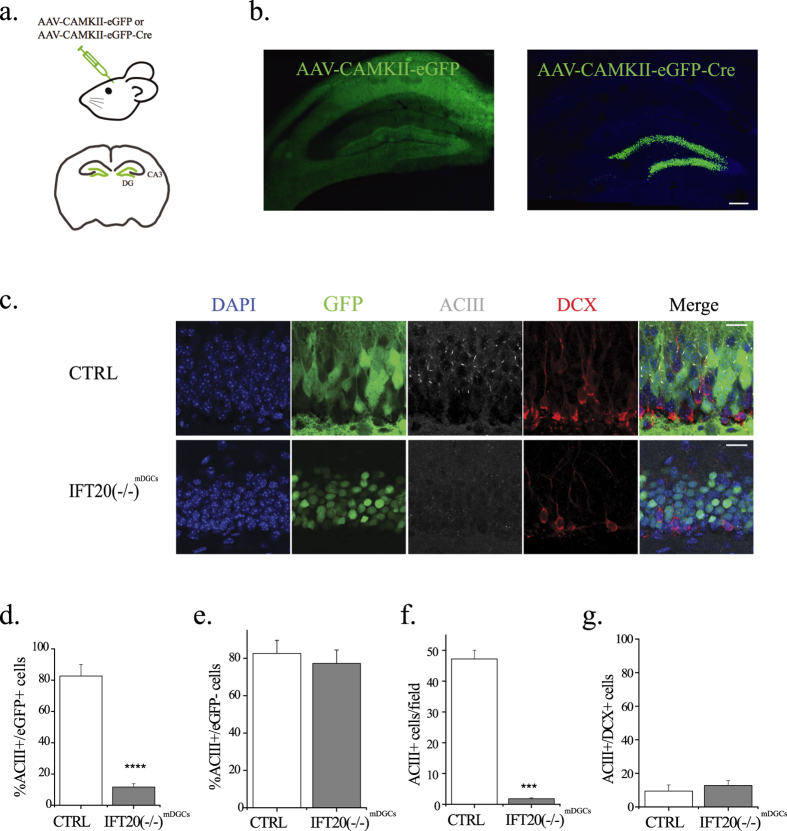
Depletion of primary cilia from mature dentate granule cells. (**a**) Schematic diagram of the AAV injection in the dentate gyrus. (**b**) Representative confocal images of the hippocampus of IFT20 fl/fl mice transduced with AAV-CaMKII-eGFP or AAV-CaMKII-eGFP-Cre virus at 28 days post AAV labeling. Scale bar: 200 μm. (**c**) Representative confocal images of the granule cell layer of AAV-CaMKII-eGFP or AAV-CaMKII-eGFP-Cre 28 days post injection. Immunostaining with anti DCX (red) and ACIII (white) reveals that primary cilia were selectively ablated in mature DGCs by AAV-CaMKII-eGFP-Cre transduction, whereas no ciliary disruption was found by AAV-CaMKII-eGFP transduction. Scale bars: 30 μm (**d**) AAV-CaMKII-eGFP-Cre transduced cells exhibited a significantly depressed rate of ACIII expression versus AAV-CaMKII-eGFP controls. (Cre: 11.7 ± 2.1% ACIII+, eGFP: 82.6 ± 7.4% ACIII+; two-tailed un-paired t-test p = 8.5 × 10^−7^). **(e)** Cells in the granule cell layer that did not take up virus expressed primary cilia at comparable rates between eGFP control and Cre groups (CTRL: 82.6 ± 7.0%; Cre: 77.3 ± 7.2%; two-tailed unpaired t-test p = 0.634; n = 3,3). **(f)** Cre transduction resulted in significant gross depletion of primary cilia. (Averaged number of ACIII positive cells per field in CTRL and IFT20(−/−)^mDGCs^: 47.23 ± 2.78 for CTRL and 1.8 ± 0.28 for IFT20(−/−)^mDGCs^; p = 5.985 × 10^−15^; n = 4,4). **(g)** Expression of ACIII in DCX+ cells did not differ significantly between eGFP-Cre and eGFP controls (CTRL: 9.5 ± 3.6% co-localization; IFT20(−/−)^mDGCs^ : 12.8 ± 2.9% co-localization; two-tailed unpaired t-test p = 0.50). Fields size: 320 μm × 320 μm × 36 μm. ***p < 0.001; ****p < 0.0001; n is the number of animals.

We set out to examine the physiological and behavioral role of primary cilia in mature DGCs by specifically depleting IFT20 in these cells. AAV9 expressing Cre recombinase under the control of the CaMKII promoter (or eGFP under the CaMKII promoter as control) was stereotaxically injected into the dentate gyrus of adult floxed IFT20 mice^22^ (Fig. 1a). Two weeks after the injection, we observed extensive transduction of AAV along the entire anterior/posterior axis of the dentate gyrus with the expression of the CaMKII promoter driven Cre recombinase, with 86.3 ± 3.2% of cells in the granule cell layer expressing Cre (as seen by eGFP positivity), or eGFP alone, with 89.4 ± 7.0% of cells in the granule cell layer labeled with eGFP in the control condition (Fig. 1b,c). We noted that in the control group there was wide spread of the GFP signal into other hippocampal regions. This may result from better diffusion of the AAV-eGFP, which remains to be determined. However, labeling in the Cre group was much more specific to the DG because Cre-eGFP was constrained to the nucleus of infected cells in the experimental condition (Fig. 1b). To confirm the specificity of viral transduction to the granule cell population, we co-stained transduced cells for parvalbumin (PV), a marker of the major population of interneurons in the DG, in mice injected with AAV-CaMKII-eGFP and AAV-CAG-eGFP. We observed no overlap between PV+ cells and GFP+ cells in the CaMKII group (0/100 cells). As expected, as a comparison, the AAV-CAG (a generic promoter)-eGFP injected group exhibited ~27% of PV+ cells that co-localized with GFP (14/51 cells) (Supplementary Fig. 1).

To confirm sufficient ablation of primary cilia in AAV-CaMKII-eGFP-Cre-infected cells, we quantified the number of GFP+ cells harboring the ciliary protein ACIII. As expected, the vast majority of Cre+ cells lacked expression of ACIII, compared to the proportion of eGFP+ cells expressing ACIII in control mice (Fig. 1d). By contrast, the small minority of cells in the granule cell layer that were Cre negative (13.7 ± 3.2%) expressed ACIII at levels comparable to those observed in the small minority (10.6 ± 7.0%) of eGFP negative control cells (Fig. 1e). Likewise, the total number of ACIII+ cells was dramatically reduced in Cre-expressing animals (IFT20(−/−)^mDGCs^) versus eGFP-controls (Fig. 1f). These results demonstrate the efficacy of our cilia ablation approach.

Given that the adult hippocampus continuously generates new dentate granule cells^23^,^24^,^25^ and that the CaMKII promoter becomes active in adult-born, mature neurons^26^, to confirm primary cilium depletion was restricted to the population of mature DGCs in designed experiments, we immunostained hippocampal sections with doublecortin (DCX), a marker of immature neurons^27^. Two weeks post injection, we observed no overlap between DCX+ and Cre-GFP+ cells in the dentate granule cell layer (Fig. 1c). To confirm that primary cilia ablation was restricted to mature but not young DGCs, we analyzed co-expression of DCX and ACIII and found no significant difference in ACIII expression between IFT20(−/−)^mDGCs^ and controls (Fig. 1g). To determine whether there was any effect of cilia depletion in mature DGCs on hippocampal neurogenesis, we counted DCX+ cells in the granule cell layer and analyzed their dendritic morphology. We found that the number of DCX+ cells was similar between the Cre and GFP only expressing groups, and that there were no significant differences in dendrite morphology as measured by Sholl analysis, suggesting that cilia depletion in mature DGCs did not grossly disrupt adult neurogenesis (Supplementary Fig. 2). Together, our results indicate that AAV-mediated Cre expression in IFT20 floxed animals is an efficient method for ciliary deletion from a large portion of mature DGCs, which is likely sufficient for testing behavioral and physiological functions of primary cilia.

### Depletion of primary cilia from mature DGCs impaired hippocampus-associated contextual fear memory and spatial memory but not pattern discrimination or anxiety-like behaviors

**Figure 2:**
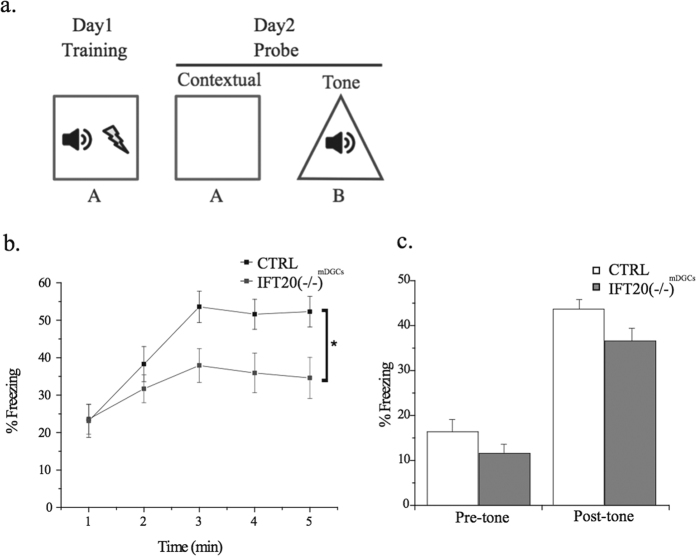
Primary cilia ablation from mature dentate granule cells impairs contextual fear memory. (**a**) Experimental design for CFC test. (**b**) IFT20(−/−)^mDGCs^ mice showed a decreased freezing response on the CFC test (repeated measures ANOVA main effect of condition: F(1,16) = 6.979, p = 0.018; n = 17,19). (**c)** IFT20(−/−)^mDGCs^ and CTRL mice did not differ significantly on the tone-cued fear memory test (two-way ANOVA main effect of condition: F(2,69) = 2.444; p = 0.094; n = 17,19). *p < 0.05; n is the number of animals.

To examine whether depletion of primary cilia from mature DGCs affects hippocampal behaviors, we focused on testing memory by performing a contextual fear memory test. We arranged two groups of animals as described in Fig. 1 and performed the CFC test as illustrated in Fig. 2a. We found that the freezing level in context A was significantly lower in IFT20(−/−)^mDGCs^ as compared to CTRL mice (Fig. 2b). However, IFT20(−/−)^mDGCs^ mice did not differ significantly from CTRL mice on tone-cued freezing in context B (Fig. 2c), suggesting a specific defect in contextual fear memory. This set of results indicates that primary cilia in mature DGCs are involved in normal contextual fear but not in tone-cued fear memory.

**Figure 3:**
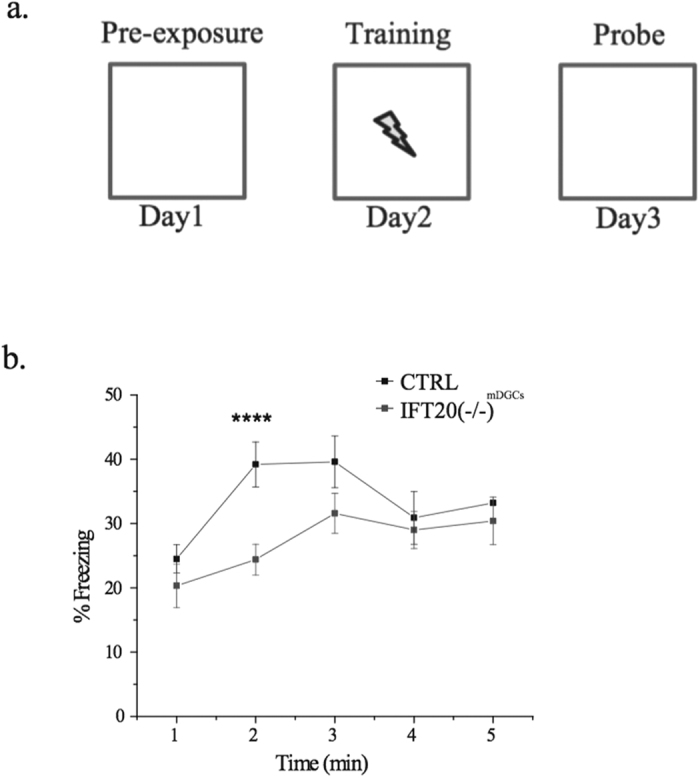
Ablation of primary cilia from mature dentate granule cells impairs pre-exposure contextual fear memory. (**a**) Experimental design for PECFC test. (**b**) IFT20(−/−)^mDGCs^ showed a reduced freezing response as compare to CTRL when re-exposed to a fear context (repeated measures ANOVA interaction between condition and time F(1, 25) = 4.747, p < 0.0001, followed by LSD comparison at 60–120 sec: p = 4.41 × 10^−5^; n = 22,23). ****p < 0.0001; n is the number of animals.

The impairments in CFC observed after the depletion of cilia from mature DGCs, together with a prior study demonstrating that these neurons mediate hippocampus-dependent contextual pattern memory^28^, led us to speculate that depletion of primary cilia from mature DGCs may affect pattern memory encoding. We therefore performed the PECFC test as previously described^17^. The experimental procedure for these tests is shown in Fig. 3a. Interestingly, we found that IFT20(−/−)^mDGCs^ mice exhibited significantly less freezing behavior as compared to CTRL mice (Fig. 3b). This finding suggests a role of primary cilia in mature DGCs in pre-exposed contextual memory.

We next explored the role of primary cilia in another DG-dependent contextual memory task, pattern encoding, the ability to discriminate distinct representations between similar contexts/episodes^18^,^29^,^30^,^31^. To examine whether primary cilia in mature DGCs are necessary for pattern encoding, we performed a contextual discrimination task. We found that IFT20(−/−)^mDGCs^ mice did not differ significantly from controls in terms of their ability to discriminate between two similar environments, indicating that primary cilia are not involved, at least directly, in pattern encoding (Supplementary Fig. 3).

In addition to its role in contextual memory, the hippocampus is highly involved in spatial information processing and memory^32^. To examine whether primary cilia in mature DGCs are necessary for spatial memory expression, we performed the spatial novelty recognition test. We found that unlike CTRL mice, IFT20(−/−)^mDGCs^ mice did not show a significant preference for an object at a novel location, as measured by exploration time at either a novel or familiar location (Supplementary Fig. 4). This suggests that primary cilia in mature DGCs are important for spatial memory expression.

The hippocampus is also known to be involved in anxiety- and depression-associated behaviors^33^,^34^,^35^. To investigate a possible ciliary function in these behaviors, we performed the elevated plus maze (Supplementary Fig. 5) and light/dark transition tests (Supplementary Fig. 6) after depleting primary cilia from mature DGCs. We found that IFT20(−/−)^mDGCs^ mice did not exhibit significant behavioral differences on either test as compared to CTRL mice, suggesting that primary cilia are not directly involved in emotional behaviors, at least as measured by these specific tests.

### Ablation of primary cilia from mature DGCs augmented long-term potentiation induced at mossy fiber synapses

**Figure 4:**
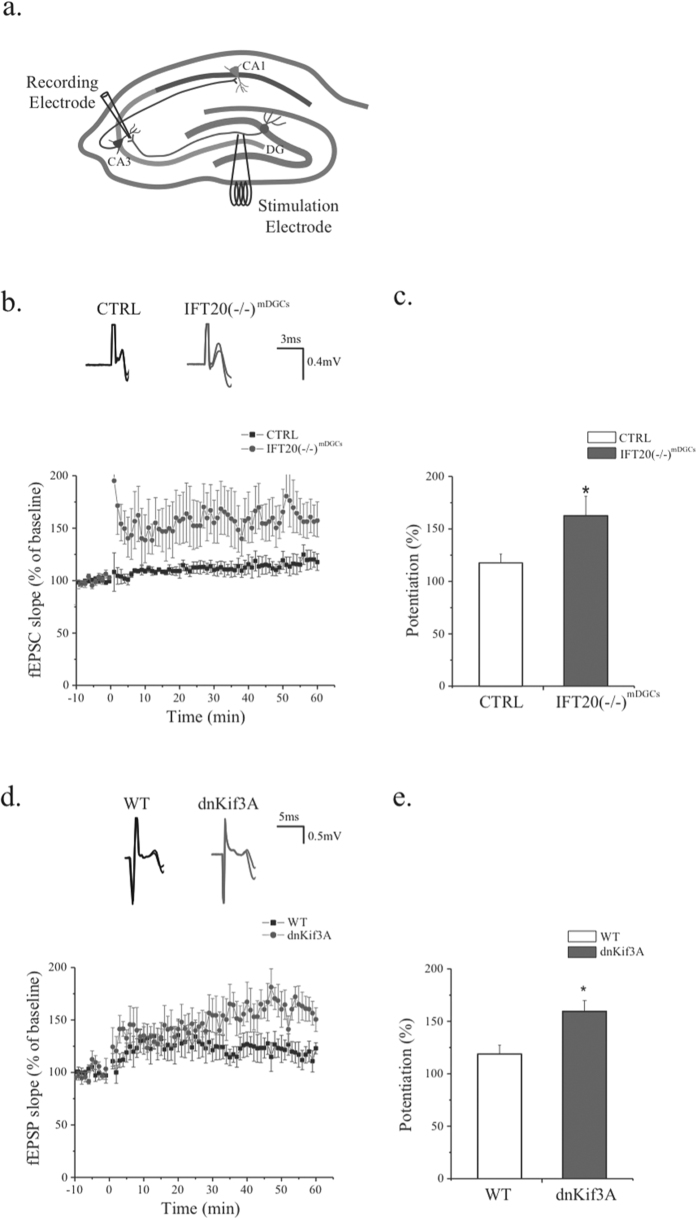
Removal of primary cilia from mature dentate granule cells increases synaptic plasticity in the mossy fiber pathway. (**a**) A schematic representation of the tri-synaptic circuit and the position of stimulating and recording electrodes. (**b**) Top, representative traces of both CTRL and IFT20(−/−)^mDGCs^ recorded before and after HFS. Bottom, IFT20(−/−)^mDGCs^ show increased LTP compare to CTRL. (**c**) Average potentiation of fEPSPs slope during 50–60 minute showing enhanced LTP in IFT20(−/−)^mDGCs^ (two-tailed unpaired t-test p = 0.048; n = 7,8). (**d**) Top, representative traces of both WT and dnKif3A recorded before and after HFS. Bottom, dnKif3A show increased LTP compare to WT. (**e**) Average potentiation of fEPSPs slope during 50–60 minute showing LTP enhancement in dnKif3A (two-tailed unpaired t-test p = 0.048; n = 5, 4). *p < 0.05; n is the number of animals.

The defect in contextual memory observed after depletion of cilia in mature DGCs suggests a potential impairment in the function of MF synapses of these neurons, as mature, adult-born DGCs contribute importantly to LTP at these synapses^36^,^37^. We therefore recorded fEPSPs and studied plasticity by inducing LTP in area CA3 of acutely prepared hippocampal slices while stimulating the MFs (Fig. 4a). To determine whether baseline synaptic transmission was affected we performed an input-output (I-O) analysis of fEPSPs in CTRL and IFT20(−/−)^mDGCs^ mice, showing similar I-O curves as stimulation intensity was increased from 10 to 35 mA (Supplementary Fig. 7). We next induced LTP by high frequency stimulation as we previously described^16^. Interestingly, MF synapses in the CA3 of IFT20(−/−)^mDGCs^ mice exhibited increased LTP as compared to those of CTRL mice (Fig. 4b). The amplitude of potentiation measured from 50 to 60 minutes after the stimulation was 117.75 ± 8.25% for the CTRL group and 163.69 ± 18.59% for the IFT20(−/−)^mDGCs^ group (p = 0.048; n = 7,8; two-tailed unpaired t-test; Fig. 4c).

Several recent studies have shown that IFT20 potentially has non-ciliary functions in certain cell types^38^,^39^,^40^. To confirm that the enhanced LTP after cilia depletion was indeed due to the ciliary function of IFT20, we knocked down another ciliary protein, Kif3A, which affects cilium formation as we and others recently reported^9^,^41^. We induced the expression of a dominant negative Kif3A (dnKif3A) in mature DGCs via co-injection of AAV9-CaMKII-Cre and AAV9-DIO-dnKif3A-dTomato into the dentate gyrus of wild type (WT) mice. Similar to what we observed in IFT20(−/−)^mDGCs^ mice, we found that mice expressing dnKif3A in mature DGCs exhibited increased LTP above levels observed in WT (117.75 ± 8.25% of baseline for the WT and 163.69 ± 18.59% of baseline for the dnKif3A; p = 0.048; n = 5,4; Fig. 4d,e). Together, this set of results suggests a potential role of primary cilia in synaptic plasticity at the MF pathway of mature DGCs.

## Discussion

In this study, we deleted IFT20 or disrupted the function of Kif3A to impair ciliary stability^42^,^43^ of mature DGCs to decipher the function of primary cilia in hippocampal physiology. Conditionally knocking out IFT20 in mature DGCs, we observed defects in contextual fear memory and pre-exposure contextual fear memory expression, as evidenced by decreased freezing behavior upon exposure to a previously encountered fear context. Importantly, anxiety-like behaviors were unaffected after cilia depletion, demonstrating that differences in basal anxiety-like states are unlikely to account for the observed differences in contextual fear memory expression. We also observed a deficit in spatial recognition memory, as evidenced by a lack of preferential exploration of a novel object location in IFT20(−/−)^mDGCs^ mice. On the other hand, contextual pattern separation abilities were spared, implicating primary cilia as important in some, but not all forms of hippocampus-dependent contextual memory. Finally, we discovered a substantial abnormality in the plasticity of MF synapses after cilia depletion, which may account for the behavioral impairments observed. In sum, our data suggest a function of mature DGCs’ primary cilia in hippocampus-dependent behavior and plasticity.

Though the major downstream signaling pathways of primary cilia that lead to the observed disruptions remain to be determined, the Wnt/β-catenin pathway is a possible candidate, given previous findings by others and us^9^,^44^. Moreover, primary cilia are enriched with several G-protein couple receptors including somatostatin receptor 3 (SSTR3), serotonin receptor subtype 6 (5-HT_6_) and enzymes including ACIII, and signaling pathways downstream of these could be another possible cause. Indeed, in SSTR3 or ACIII-knockout animals, several hippocampus-dependent behavioral defects have been identified^13^,^14^. However, given that these tests have used animals with constitutively knocked out genes, it has been challenging to conclude whether these defects were the result of disruptions during development. Moreover, because both manipulations caused ciliary loss, it remains unknown whether these defects resulted from the specific loss of SSTR3 or ACIII, or from the loss of primary cilia *per se*. Likewise, more work will be needed to definitively confirm that the behavioral effects observed in the current study are not the result of non-ciliary functions of the proteins we disrupted.

Learning and memory generally occur via a three-step process: encoding, consolidation, and retrieval. The DG-CA3 juncture has been found to be heavily involved in the formation of a contextual memory representation^29^,^45^, which is consistent with our finding after cilium depletion in mature DGCs. Our observation is further strengthened upon finding a defect in plasticity induced in area CA3. Previously, it was reported that functional ablation of SSTR3 resulted in aberrant LTP at the Schaffer collateral synapse^14^. Here, we instead tested LTP in the target area likely responsible for contextual memory expression and found a substantial increase in LTP amplitude. Given the unchanged basal transmission, we assume that this likely resulted from altered plasticity factors such as NMDA receptor subunit composition or expression level^7^, however this requires further investigation. In any case, synaptic plasticity has been considered a cellular mechanism of memory encoding^46^ and memory recall during pattern completion^47^,^48^,^49^. After cilium depletion from mature DGCs, we found augmented plasticity in CA3 synapses. Although we are uncertain whether potentiated plasticity directly caused memory defects, given the role of the tri-synaptic circuit in memory^50^, the altered plasticity can at least partially explain the memory deficits. However, a novel approach to downgrade the enhanced plasticity will be helpful to address this confusion.

Our finding that depletion of IFT20 in mature DGCs leads to impairments in both CFC and PECFC suggest that primary cilia are important for proper rapid memory encoding. A potential model consistent with these findings is that primary cilia in mature DGCs may regulate the activity of mature DGCs and their CA3 output^51^, facilitating the rapid encoding of incoming contextual information. Furthermore, it is possible that primary cilia in mature DGCs indirectly modulate the CA3 local circuit activity to influence memory encoding. Both possibilities will require further exploration.

Finally, one potential limitation that we should point out is the viral vector-based approach for targeting a specific cell population, although this approach is still currently widely used in opto- and chemo-genetic gene delivery. In this study, we expressed Cre recombinase under the CaMKII promoter to target DGCs. Although we observed robust targeting of CaMKII-driven Cre expression to DGCs and noted no overlap between PV+ interneurons and CaMKII-driven GFP expression in the dentate gyrus, others have reported limited specificity of this promoter to excitatory neurons in cortical layer V and hippocampal area CA1, labeling some inhibitory PV+ interneurons as well^52^,^53^. In our study, we did identify a small amount of Cre expression in the hilus, thus we cannot entirely exclude a potential contribution of a small proportion of hilar mossy cells to our findings. Furthermore, there may have been a small population of interneurons also labeled. However, we conclude that the expression of Cre must have been very low since we detected no fluorescent protein nor ciliary defect. Nevertheless, more specific promoters and MiniPromoters should be explored to facilitate virus-based genetic manipulations, which will be a great complementary technology to the transgenic approach.

In the adult brain, new DGCs are continuously generated^23^,^24^,^25^. Several studies have demonstrated that young, immature neurons play distinct roles from those of mature neurons^18^,^28^,^31^,^54^. Manipulating mature DGCs through their primary cilia may disrupt the balance of activity between young and mature DGCs in the dentate gyrus by decreasing the neuronal activity of mature DGCs specifically. Since young DGCs are important for pattern separation^31^ and we observed no impairments on such a behavioral assay, our findings are consistent with the idea that mature, but not immature DGC activity, is disrupted upon cilia depletion, however further work will be required to confirm this idea. A potential increase in activity and plasticity of young neurons relative to mature neurons could explain our finding of enhanced MF LTP in IFT20(−/−)^mDGCs^ and dnKif3A-expressing mice.

In summary, we demonstrated a physiological function of the neuronal primary cilium. Our findings implicate primary cilia of mature DGCs in hippocampus-associated contextual memory. It will be interesting to further test the cellular mechanisms by which primary cilia regulate the activity of the dentate granule neurons, and also to examine possible effects of primary cilia in other brain regions.

## Additional Information

**How to cite this article**: Rhee, S. *et al*. Depletion of primary cilia from mature dentate granule cells impairs hippocampus-dependent contextual memory. *Sci. Rep.* **6**, 34370; doi: 10.1038/srep34370 (2016).

## Supplementary Material

Supplementary Information
