# Supplementary material for: Depletion of primary cilia from mature dentate granule cells impairs hippocampus-dependent contextual memory
Source: Sci Rep. 2016 Sep 28;6:34370. doi: 10.1038/srep34370 (PMC5039642; doi:10.1038/srep34370)
Supplement: Supplementary Information [file srep34370-s1.pdf]

Depletion of primary cilia from mature dentate granule cells impairs hippocampus-dependent contextual memory

Soyoung Rhee[1*], Gregory W. Kirschen[1,2*], Yan Gu[3, 4], Shaoyu Ge[4#]

[1]Program in Molecular and Cellular Pharmacology. State University of New York at Stony Brook, Stony Brook, New York, USA. [2]Medical Scientist Training Program. State University of New York at Stony Brook, Stony Brook, New York, USA.[3]Center of Stem Cell and Regenerative Medicine, Zhejiang University School of Medicine, Hangzhou, China. [4]Department of Neurobiology and Behavior, State University of New York at Stony Brook, Stony Brook, New York, USA.

[*] These authors contributed equally to the work.

[#]Correspondence should be addressed to Shaoyu Ge (shaoyu.ge@stonybrook.edu)

Supplementary Figure 1. Specificity of AAV-CaMKII-driving expression in the dentate gyrus

(a) Representative image of AAV-CAG-eGFP showing co-localization of GFP and parvalbumin (PV).

(b) Representative image of AAV-CaMKII-eGFP showing lack of co-localization between GFP and PV.

Scale bars: 30μm.

Supplementary Figure 2. Adult neurogenesis was not significantly affected by removal of primary cilia

(a) The averaged number of DCX positive cells was not significantly different between CTRL and IFT20(-/-)^{mDGCs} (30.28±1.91 for CTRL and 30.51±2.34 for IFT20(-/-)^{mDGCs}; two-tailed unpaired t-test p=0.94, n=6,6). n is the number of animals; Field size: 320μm ×320μm x 36μm.

(b) Dendritic complexity of DCX+ cells did not differ significantly between CTRL and IFT20(-/-)^{mDGCs} mice (repeated measures ANOVA main effect of condition F(1,17) = 1.512; p = 0.236; n=3,3)

n is the number of animals.

Supplementary Figure 3. Ablation of primary cilia from mature dentate granule cells does not alter pattern encoding

(a) Pattern encoding task paradigm.

(b-c) Both control mice and IFT20(-/-)^{mDGCs} mice learned to discriminate between a a

shock-associated environment (A) and safe environment (B). CTRL: Repeated measures ANOVA main effect of environment: $F(1,17) = 5.935$; $p = 0.026$; interaction between environment and day: $F(7,11) = 36.163$; $p < 0.0001$; n = 13. IFT20(-/-)^{mDGCs}: Repeated measures ANOVA main effect of environment: $F(1,15) = 6.568$; $p = 0.022$; interaction between environment and day: $F(7,9) = 47.140$; $p < 0.0001$; n = 11.

(d) Average discrimination ratios (DRs) between safe and shock-associated environments did not differ significantly between groups (Repeated measures ANOVA main effect of group: $F(1,15) = 1.161$; $p = 0.298$; interaction between group and day: $F(7,9) = 2.559$; $p = 0.095$; n = 18,16).

*$p<0.05$; n is the number of animals.

Supplementary Figure 4. Ablation of primary cilia from mature dentate granule cells affects spatial novelty recognition memory

(a) Spatial novelty recognition test paradigm.

(b) CTRL mice spent significantly more time exploring a novel object location versus a familiar location, however IFT20(-/-)^{mDGCs} mice did not spend a significantly different amount of time exploring either of the object locations (CTRL: old location 21.0±2.4 sec; new location 28.4±2.5 sec; two-tailed unpaired t-test $p = 0.044$; IFT20(-/-)^{mDGCs}: old location 27.2±3.4 sec; new location 26.6±2.5 sec; two-tailed unpaired t-test $p = 0.895$; n=11,11).

(c) CTRL mice exhibited a trend toward more entries into a novel object location versus a familiar location, however IFT20(-/-)^{mDGCs} mice did not (CTRL: old location 162.0±19.7 entries; new location 214.6±24.7 entries; two-tailed unpaired t-test $p = 0.111$; IFT20(-/-)^{mDGCs}: old location 200.0±28.9 entries; new location 196.8±21.0 sec; two-tailed unpaired t-

test p = 0.930; n=11,11).

n is the number of animals.

Supplementary Figure 5. Ablation of primary cilia from mature dentate granule cells does not alter anxiety-like behavior on the elevated plus maze

Average time spent at center, open and closed arms during the experiment. No significant difference between CTRL and IFT20(-/-)^{mDGCs} mice (24.80±5.14% for CTRL and 32.87±5.86% for IFT20(-/-)^{mDGCs} mice at the open arm; two-tailed unpaired t-test p=0.31; n=18,20).

n is the number of animals.

Supplementary Figure 6. Ablation of primary cilia from mature dentate granule cells does not alter anxiety-like behavior on the light/dark transition test Average time spent in light and dark compartment during 10 minutes. The time spent in dark compartment differ significantly between CTRL and IFT20(-/-)^{mDGCs} mice (80.48±2.39% for CTRL and 78.40±1.88 for IFT20(-/-)^{mDGCs}; two-tailed unpaired t-test p=0.50; n=18,20; n is the number of animals).

Supplementary Figure 7. I-O curve for CTRL and IFT20(-/-)^{mDGCs}

Input and output curve obtained from CTRL and IFT20(-/-)^{mDGCs} mice. Synaptic transmission was not altered in IFT20(-/-)^{mDGCs} from 10 to 35mA of stimulation intensity (n=5,6; n is the number of brain slices).

a.

AAV-CAG-eGFP

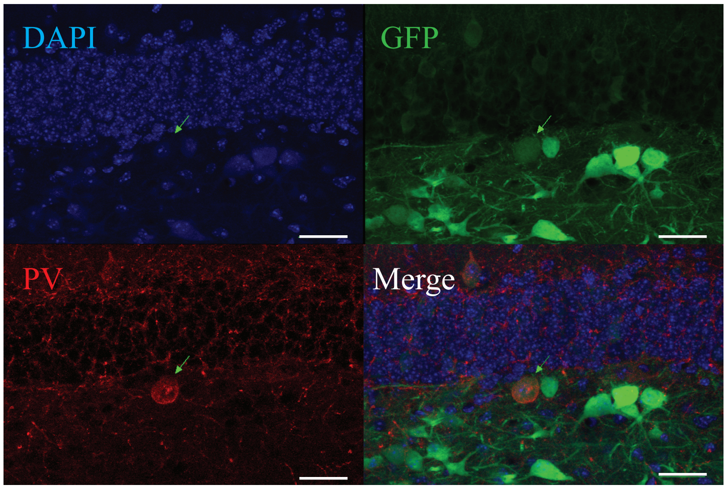

b.

AAV-CaMKII-eGFP

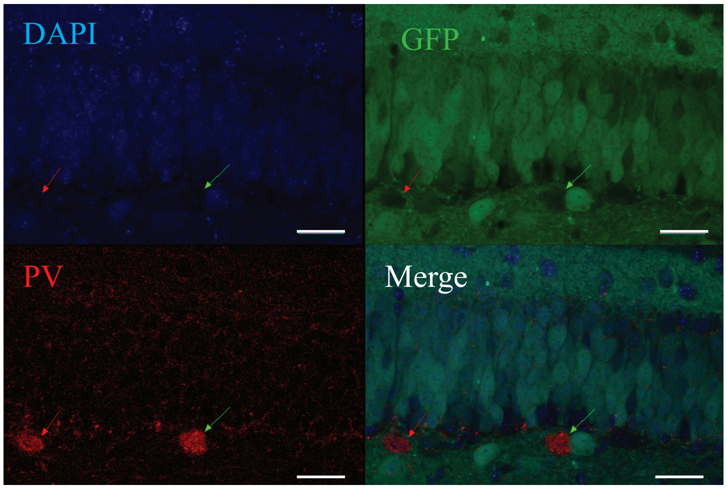

Supplementary Figure 1. Specificity of AAV-CaMKII-driving expression in the dentate gyrus

a. b.

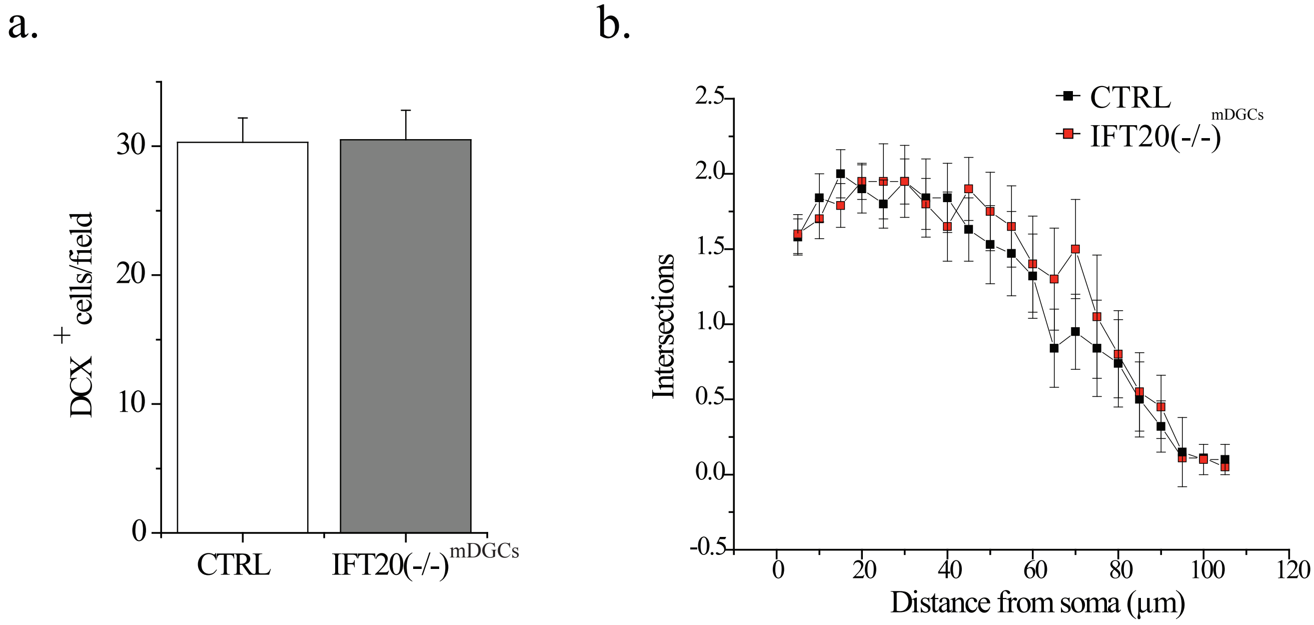

Supplementary Figure 2. Adult neurogenesis was not significantly affected by removal of primary cilia

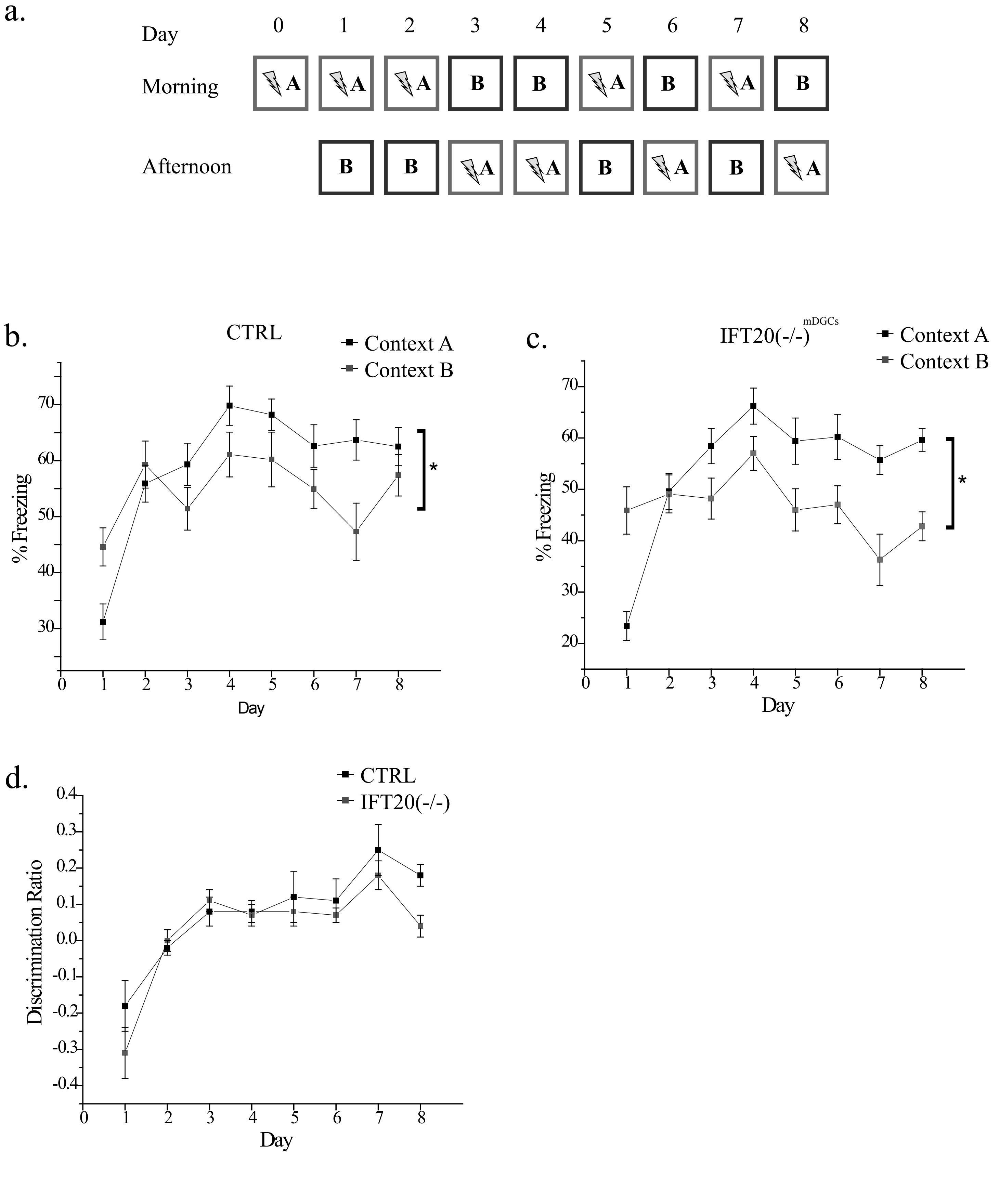

Supplementary Figure 3. Ablation of primary cilia from mature dentate granule cells does not alter pattern encoding

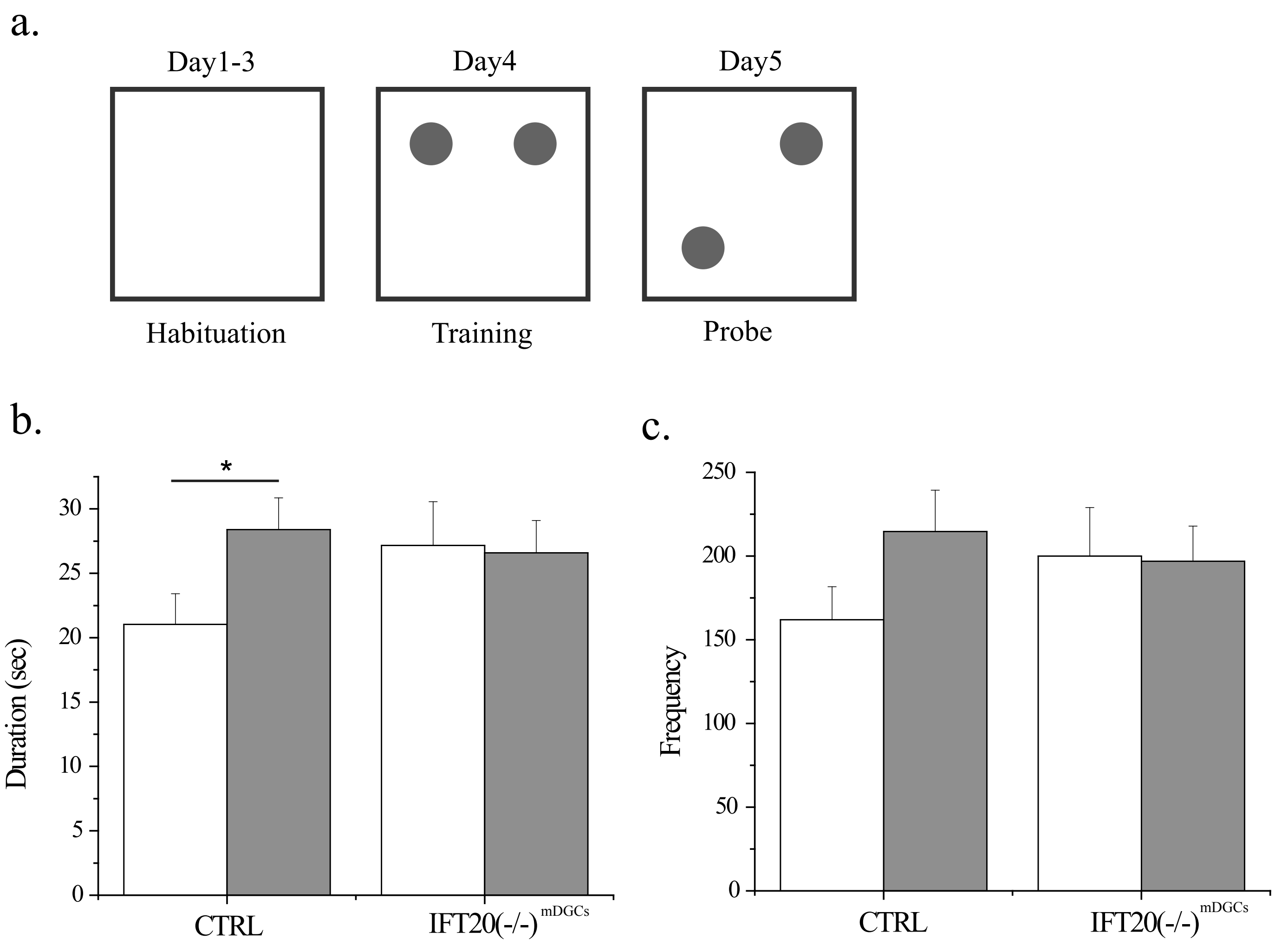

Supplementary Figure 4. Ablation of primary cilia from mature dentate granule cells affects spatial novelty recognition memory

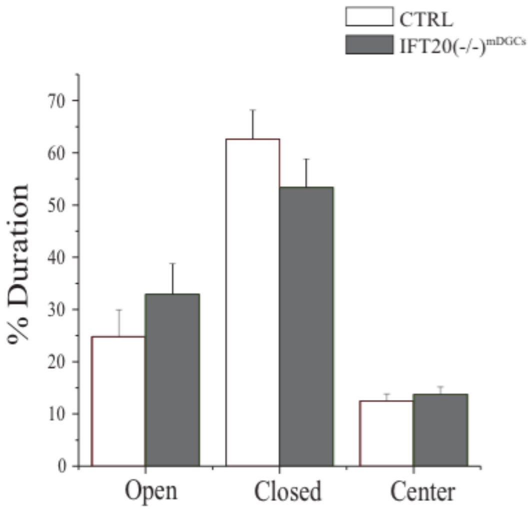

Supplementary Figure 5. Ablation of primary cilia from mature dentate granule cells does not alter anxiety-like behavior on the elevated plus maze

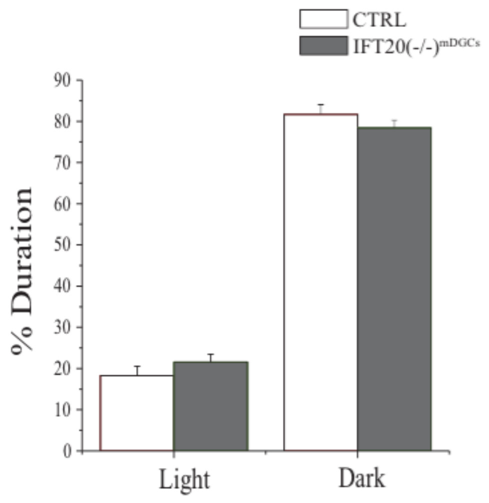

Supplementary Figure 6. Ablation of primary cilia from mature dentate granule cells does not alter anxiety-like behavior on the light/dark transition test

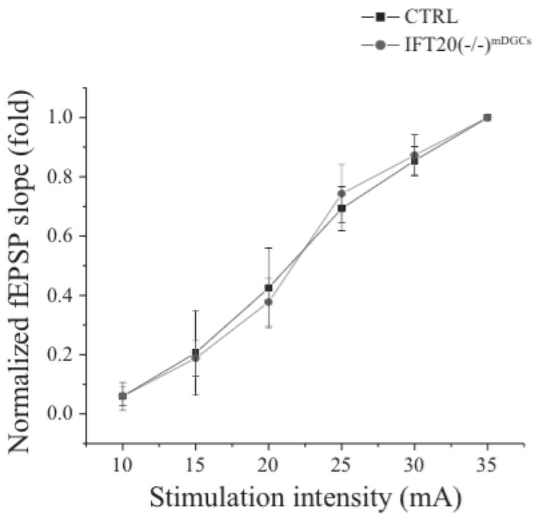

Supplementary Figure 7. I-O curve for CTRL and IFT20(-/-)^{mDGCs}
